# Managerial practices and factors influencing reproductive performance of dairy cows in urban/peri-urban areas of Kampala and Gulu, Uganda

**DOI:** 10.1186/s13028-015-0122-2

**Published:** 2015-06-25

**Authors:** Kanyima M. Benon, David O. Owiny, Renée Båge, Maria G. Nassuna-Musoke, Patrice Humblot, Ulf Magnusson

**Affiliations:** College of Veterinary Medicine, Animal Resources and Biosecurity, Makerere University, P. O. Box 7062, Kampala, Uganda; Division of Reproduction, Department of Clinical Sciences, Faculty of Veterinary Medicine and Animal Sciences, Swedish University of Agricultural Sciences, P.O. Box 7054, SE-750 07 Uppsala, Sweden

**Keywords:** Urban peri-urban farming, Dairy cow, Milk production, Uganda, Tropics, Reproductive performance, Bovine

## Abstract

**Background:**

Urban/peri-urban dairy production and sales has evolved as an adjustment to cope with food security and economic needs for urban dwellers in low-income countries and created an opportunity to transform from subsistence rural lifestyles of dairy farming to commercial engagement in towns. However, urban/peri-urban dairy farms differ in challenges from rural dairy farms and reproduction is important and critical for assuring sustainable economic output in both environments. Here we recorded for the first time differences between two geographically and economically different cities corresponding to different settings within the same country in managerial factors influencing reproductive performance in urban/peri-urban dairy cowherds.

**Results:**

The urban/peri-urban dairy farmers in the capital Kampala were more often male (*P* = 0.002) and commercialized (*P* = 0.0025), more experienced (*P* = 0.0001) and practiced zero-grazing more often (*P* = 0.05) than in the regional municipality Gulu. Also, the milk production per herd and cow (*P* = 0.0005) and calving rate were (*P* = 0.0001) higher in Kampala and artificial insemination was more commonly (*P* = 0.002) used than in Gulu. There was no difference in abortion nor neonatal mortality rate between the two locations. Overall, calving rates were higher (*P* = 0.0003) in smaller (≤3 dairy cows) and open grazing (*P* = 0.003) herds. Abortion rates were higher among dairy herds practicing late (≥5 months) (*P* = 0.003) calf weaning and in herds with commercial purposes (*P* = 0.0001). Neonatal calf mortality was lower (*P* = 0.01) in small herds.

**Conclusion:**

The study showed significant differences between Kampala and Gulu in reproductive performance and related husbandry factors for cows in the urban/peri-urban dairy farming systems. For several reproductive performance traits we found associations with husbandry and production traits, which should be taken into account when providing advice to the urban and peri-urban dairy farmers in the tropics.

## Background

The rapid urbanization in the developing world has raised concerns about global food security in the urban areas [1]. In sub-Saharan Africa, demographic predictions indicate a tremendous urban population growth rate [2]. This in turn implies that various forms of urban agriculture will be relied upon as essential transitional strategies for feeding and employment of rural–urban immigrant populations in this region [3]. Uganda is experiencing such a rapid rural–urban migration in the two major urban centers, Kampala in the central region and post-conflict Gulu in the north. In and around both these two cities, dairy production and sale have evolved as adjustments to cope with food security and economic needs for farmers [4]. The capital city Kampala has a steady, 20-year history of urban/peri-urban (UPU) dairy production while for Gulu which has been repeatedly affected by massive rural–urban population influx resulting from civil strife, milk production is much more recent. Urban/peri-urban farming, often defined as farming taking place in a town or city and in the immediate area surrounding the city, has also created an opportunity to transform from subsistence rural lifestyles of dairy farming to commercial engagement [4–6]. However, UPU dairy farming differ in challenges from rural dairy farming, like lack of or poor quality feed, non-supportive policy environment and extension service and poor management skills among the farmers [7].

Reproduction is important and critical for assuring sustainable economic output in high producing dairy systems [8–11] as well as in low intensive dairy systems in sub-Saharan Africa [12–16]. However, necessary data on reproductive performance and the factors influencing it in sub-Saharan African UPU dairy farming is scanty. Such basic information is necessary for the development of herd health programs customized for increased productivity. This study recorded for the first time differences between two geographically and economically different settings within the same country in managerial factors influencing reproductive performance in UPU dairy cowherds. Also, overall analyses of data from the two settings showed associations between reproductive traits and socioeconomic, husbandry and production factors.

## Methods

### The study areas

Kampala, the capital city of Uganda covering some 190 km^2^ and with a population of 1,300,000 people and Gulu, a regional municipality, covering about 55 km^2^ and having a population of 150,000 are the main urban centers in the central and northern regions of Uganda, respectively. There has been rapid population growth due to rural–urban migration in Kampala in the last 25 years for economic reasons and Gulu in the last 15 years for security reasons. A cross sectional survey of cattle-keeping households engaged in dairy farming within a maximum radius of 25 Km of Kampala city center (00°18′49″N: 32°34′52″E) and Gulu municipality center (2°46'48 N: 32°18'00E), was conducted from January to July, 2011 for Kampala and July to November, 2010 for Gulu.

### Study households

**Fig. 1:**
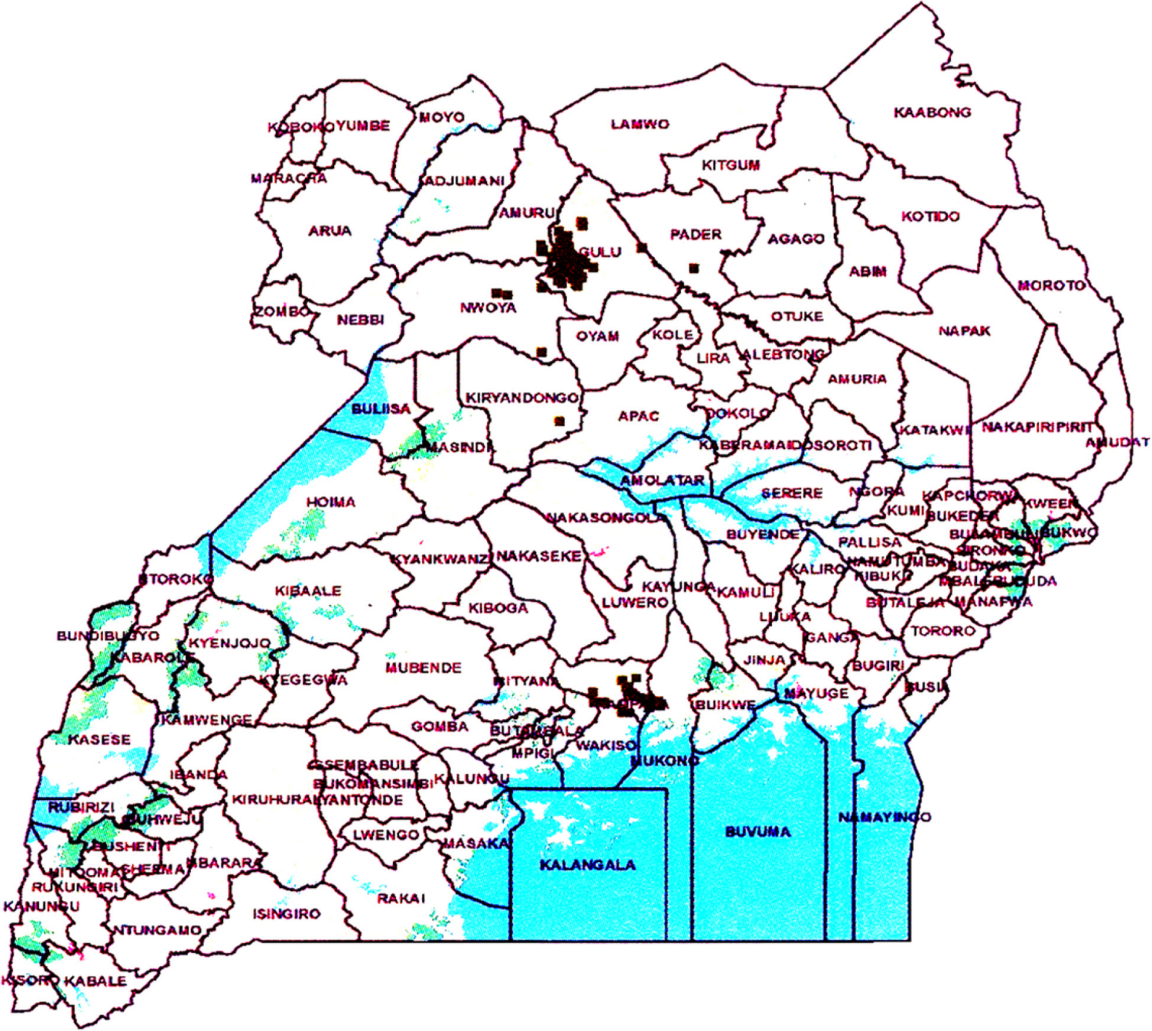
Map of the study areas in Gulu and Kampala, Uganda

Dairy farming households were selected by convenience from typical dairy farmers where appropriate data could be collected in UPU Kampala and Gulu. Data on household socio-economic position, the geographical locations (Fig. 1), dairy cow husbandry, herd management practices and reproductive performance were collected at household visits by direct questioning, discussion and observations using a structured protocol and a pre-designed questionnaire. The visits in Kampala were performed by one artificial insemination technician, a veterinary student or the first author (BMK) and in Gulu by two field veterinarians, three husbandry officers or two artificial insemination technicians. All animals included in the study were treated according to the ethical standards of Makerere University. The farmers were informed about the purpose of the study and their oral consent was sought prior to their participation in the study.

### Study variables

#### Cow husbandry

Data included farmer address, role (owner or other), level of education (no school up to primary, post-primary), gender (male, female), farming experience (0–2, 3–5, >5 years), membership to farmer-organization (no, yes), main objectives of farming (commercial, subsistence or mixed) and location of the household by Global Positioning System (GPS). Data on cow husbandry included production system (zero or open grazing), existence of infrastructure for cows (corrals, cattle housing, stocks and tick control facilities (yes/no), farm records (health, finance, production), herd size (1–2,3-8, or >8 cows; in the analysis of variance only two classes were used: 3 ≤ and 3>), total milk (litres) produced per farm per day (≤5, 5.1-10, >10 l), average milk produced per cow per day (≤2, 2.1-10, >10 l) on the day before the interview, and use of feed supplements (dairy meal, banana peels and crop residues (yes/no)). Also recorded were breeding methods (AI; natural service), and age at weaning (2–3, 4, ≥5 months). Background of the person that most often handled cases of reproductive disorder (trained or untrained) and farmers’ opinion about the desirable relative haste to manually remove retained foetal membranes (≤3 h, >3 h) after calving, were recorded as a proxy of the adoption of traditional pastoralists practices (where an early removal is common).

#### Cow reproductive performance

Data regarding calving rate was calculated based on the number of calves born alive per cow in in the herd one year prior to the interview. Abortion rate was calculated as proportion of offspring reported to have been lost before their expected day of parturition one year prior to the interview. Similarly, the neonatal mortality rate was calculated as a proportion of calves born alive in the year prior to the interview, but reported as having died within one month of their birth. Three classes were created for calving rate, (<33, 33–66, >66 %) and 2 classes (<10 % and ≥10 %) for abortion and neonatal calf mortality rates. All records were based on estimations by the farmer at the interview.

### Statistical analysis

Dependent reproduction variables are presented in Table 2 and the factors included in the initial analysis of dependant variables in Table 1. Relationships between variation factors and dependant variables were tested in a first step by Chi^2^ test (SAS® 9.2 software, SAS Institute Inc., Cary, NC, USA). In this step, potential links among the different variation factors were also studied by Chi^2^ test. Following the analysis of the Chi^2^ matrix, variation factors associated with the dependant variables at a threshold of 20 % were introduced with their interactions in multivariate models by ANOVA (SAS, Proc. GLM). Models were run on the original dependant variables and following arc sinϒp transformation. The multivariate models were simplified step by step to keep only variation factors and significant corresponding interactions at the threshold of 10 %. All analyses where performed following weighting of percentage for the number of female animals present in each herd and transforming data to improve normal distribution. Following ANOVA, the Scheffe test option was used in the case of multiple comparisons (more than 2 means compared). The specific effect of herd size on conception rates was further studied by non-parametric analyses (Kruskal Wallis, Wilcoxon and Van Waerden; SAS, Proc. NPAR1). Herd size was included as a factor either from non-transformed number of cows and following distribution of herds in 7 herd classes with more than 25 herds per class. Differences with *P* values <0.05 were considered significant and differences with p-values between *P* < 0.05 and 0.10 reported as tendencies.

**Table 1:** Comparison (Chi^2^) of socio economic and cow husbandry factors for dairy cattle-keeping households in urban and peri-urban Kampala and Gulu

Socio-economic factors and cow husbandry variables	Frequency (%) distribution per location		*P*-value
	Kampala	Gulu	
Role of cow caretaker in the farm			>0.15
Owner	83	87	
Other	17	13	
Education levels of respondent/owner			0.002
No school to primary	42	64	
Post-primary	58	36	
Gender of cow caretaker			0.002
Male	77	55	
Female	23	45	
Farming experience			0.0001
0-2 years	2	33	
3-5 years	44	37	
>5 years	54	30	
Farmer membership to farmer organization			0.001
No	59	36	
Yes	41	64	
Farming purpose			0.0025
Commercial	61	39	
Subsistence or subsistence-commercial mix	39	61	
Farming system			0.05
Zero grazing	77	64	
Open grazing	23	36	
Established farm structure for management			0.0001
Yes	98	45	
None	2	55	
Farm record keeping			
Records kept	98	56	0.0001
No records	2	44	
Herd size			0.08
<3 cows	58	42	
3-8 cows	23	31	
>8 cows	19	27	
Milk produced at farm per day (total milk)			0.0005
0-5 l	5	16	
5.1-10 l	19	35	
>10 l	76	49	
Milk produced per cow per day			0.0001
0-2 l	2	26	
2-10 l	61	62	
>10 l	37	12	
Use of feed additives			>0.15
Yes	39	46	
No	60	54	

## Results

### Descriptive statistics

There were 64 farms in Kampala and 188 farms in Gulu providing data to the study. Dairy farming households in Kampala on average owned 5 dairy cows as compared to 2 cows in Gulu. In Kampala, herds with less than 3 cows, 3 to 8 cows and more than 8 cows were 37, 15 and 12 respectively, whereas these numbers were 78, 59 and 50 in Gulu (Table 1).

### Differences in socio-economic factors and dairy husbandry between Kampala and Gulu

The educational level among cow owners was higher (*P* = 0.002) and the care for dairy cows was more of a male than female activity (*P* = 0.002) in Kampala compared with Gulu (Table 1). In addition, dairy farmers in Kampala were more (*P* = 0.0001) experienced (≥3 years), but belonged less (*P* = 0.0001) to farmer organization than in Gulu. Engagement into dairy farming was more commonly commercial (*P* = 0.0025) in Kampala rather than a subsistence or subsistence-commercial ‘mix’ engagement as in Gulu. More zero than open grazing (*P* = 0.05) was practiced in Kampala than Gulu. Farms with established farm structures for dairy cow management were more common (*P* = 0.001) in Kampala than in Gulu as were farms with record keeping (*P* = 0.0001). There was a tendency towards a higher frequency of large herds (*P* = 0.08) in Gulu compared with Kampala (Table 1). The distributions of the herds for daily total milk production per farm were different (*P* = 0.0005) in Kampala and in Gulu (with more herds in the highest class of production in Kampala). This was associated with a higher proportion of dairy cows producing more than 10 l of milk/day in Kampala when compared to Gulu (*P* = 0.0001).

### Differences in reproductive management practices between Kampala and Gulu

Artificial insemination was more commonly used (*P* = 0.002) than natural service to breed dairy cows in Kampala compared with Gulu (Table 2). Farm managers rather than the trained veterinary personnel treated reproductive disorders in Kampala (*P* = 0.0001) compared with Gulu. Retained foetal membranes from dairy cows were more often manually removed within 3 h post calving (*P* = 0.0001) in Kampala than in Gulu. The distributions of herd calving rates were different (*P* = 0.0001) in Kampala and in Gulu with more herds having a calving rate >66 % and fewer herds having a calving rate <33 % in Kampala. There were no differences in the distribution of neither abortion nor neonatal mortality rates between the two locations.

**Table 2:** Comparison (Chi2) of reproductive management practices and reproductive performance for dairy cattle herds in urban/peri-urban Kampala and Gulu

Herd fertility indices and reproductive management practices	Frequency (%) distribution per location		*P*-value
	Kampala	Gulu	
Breeding method in use			0.002
Artificial Insemination (AI)	78	56	
Natural service	22	44	
Background of person handling reproductive disorders			0.0001
Veterinary personnel or officers	31	67	
Farm manager or other	69	33	
When retained placentas are handled			0.0001
By 3h after calving if seen	98	44	
Beyond 3h after calving	2	56	
Herd-level calving rates			0.0001
0-33 %	8	38	
33.1-66 %	28	46	
>66 %	64	16	
Herd abortion rate			>0.15
<10 %	70	64	
≥10 %	30	36	
Herd neonatal calf mortality rates			>0.15
<10 %	73	70	
≥10 %	27	30	

### Sources of variation of herd reproductive performance parameters

#### Calving rates

In concordance with the analyses above, the mean calving rate was higher in Kampala than in Gulu (Table 3). The main effect of farming experience was not significant. Calving rates were higher (*P* = 0.0003) in small herds (≤3 dairy cows) than in large ones. This effect of herd size was confirmed from all non-parametric tests (overall effect *P* < 0.0001). The analyzis based on herd size class, showed that herds with 1 or 2 cows had higher calving rates than herds with 3 cows or more (*P* < 0.0001). A complementary analysis based on the subset of herds having 1 (*n* = 44) or 2 cows (*n* = 71) showed that calving rates were significantly higher in one-cow herds than in herds with two cows (81 % vs. 53 %, *P* < 0.0001). However, the difference between the two classes of herd size was highly significant in Gulu (75 % vs. 48 %; *P* = 0.0007), but not in in Kampala (86 % vs. 71 %; *P* = 0.15). Dairy cow herds producing >10 l/cow/day registered higher calving rates (*P* = 0.006) than less productive ones. Calving rates were also higher (*P* = 0.003) in the open than zero grazing herds. In farms keeping no herd records, calving rates were higher (*P* = 0.04) than in those keeping records but the existence of an interaction with the production system shows that the difference exists only for open grazing herds. In Kampala, calving rates were not different if owners belonged to farmer organization unlike in Gulu where the calving rate was higher for farmers not belonging to an organization (*P* = 0.05). A significant interaction was found between farming experience and the level of production (Table 3). In farms with short farming experience (≤2 year) higher calving rates were observed with high milk production (>10 l/cow/day) whereas no difference was observed between production levels in herds with more than 2 years’ experience.

**Table 3:** Analysis of variance of calving rates for urban/peri-urban herd in Kampala and Gulu

Factor	*P*-value	Calving rate (%) (Least squares Mean ± SEM)
City	0.001	
Kampala		79.76 ± 0.05
Gulu		49.60 ± 0.04
Farming experience	>0.15	
≤2 years		67.87 ± 0.09
>2 years		61.50 ± 0.08
Herd size	0.0003	
≤3 cows		75.06 ± 0.05
>3 cows		54.30 ± 0.05
Milk production per cow	0.0061	
≤10 l/cow		49.67 ± 0.02
>10 l/cow		79.69 ± 0.09
Production system	0.0030	
Zero grazing		55.73 ± 0.05
Open grazing		73.64 ± 0.05
Record keeping	0.0380	
Yes		61.37 ± 0.04
No		67.99 ± 0.05
City * Membership to organization interaction		
Kampala * Membership	<0.0001	85.35 ± 0.05
Gulu *Membership		33.52 ± 0.06
Kampala * Non-membership	>0.15	74.17 ± 0.08
Gulu * Non membership		65.68 ± 0.05
Production system * record keeping interaction		
Zero grazing * Records	>0.15	57.34 ± 0.05
Open grazing *Records		65.40 ± 0.05
Zero grazing * No records	0.078	54.12 ± 0.06
Open grazing * No records		81.87 ± 0.06
Farming experience * Milk production per cow interaction		
Experience (≤2 y) * Production (≤10 l)	0.0001	39.49 ± 0.04
Experience (≤2 y) * Production (>10 l)		96.25 ± 0.17
Experience (>2 y) * Production (≤10 l)	0.0602	59.86 ± 0.02
Experience (>2 y) * Production (>10 l)		63.14 ± 0.04

#### Abortion rates

Abortion rates were higher (*P* = 0.003) among dairy herds practicing late (≥5 months) than earlier (2–4 months) calf weaning (Table 4). The abortion rates were lower (*P* = 0.01) among dairy herds without infrastructure for handling animals than the contrary. Abortion rates were higher (*P* = 0.0001) in herds with commercial purposes than in farms oriented towards subsistence or subsistence-commercial ‘mix’ farming. The herd size did not influence the abortion rate.

**Table 4:** Analysis of variance of abortion rate for urban/peri-urban herds in Kampala and Gulu

Factors	*P*-value	Abortion rate (%) (Least Squares Mean ± SEM)
Herd size	>0.15	
<3 cows		13.14 ± 0.02
3-8 cow		12.46 ± 0.02
>8 cows		07.02 ± 0.01
Age at weaning	0.003	
2-3 months		11.44 ± 0.02^a^
4 months		07.21 ± 0.01^b^
≥5 months		14.00 ± 0.02^c^
Farm infrastructure	0.01	
Multiple infrastructure		13.54 ± 0.01
Single or none		08.20 ± 0.01
Type of farm	0.0001	
Commercial		15.93 ± 0.01
Subsistence or subsistence–commercial mix		05.81 ± 0.01
Herd size*type of farm interaction		
<3 cows * Commercial farming	<0.13	22.71 ± 0.03
<3 cows * Subsistence or subsistence–commercial mix		03.56 ± 0.03
3-8 cows * Commercial farming	>0.15	15.50 ± 0.02
3-8 cows * Subsistence or subsistence–commercial mix		09.42 ± 0.03
>8 cows * Commercial farming	>0.15	09.59 ± 0.02
>8 cows * Subsistence or subsistence–commercial mix		04.45 ± 0.01

In case of multiple comparisons, a *vs* b *P* < 0.05 (Scheffe test)

#### Neonatal calf mortality rates

There was a strong tendency for higher (*P* = 0.06) neonatal calf mortality in herds of owners with higher education than in the herd of low/un-educated owners (Table 5). Neonatal calf mortality was lower (*P* = 0.01) in small (≤3 dairy cows) than in larger (>3 dairy cows) herds. Among the herds engaged in commercial farming, small herds (≤3 dairy cows) had lower (*P* = 0.03) neonatal calf mortality than large herds (>3 dairy cows).

**Table 5:** Analysis of variance of neonatal calf mortality for urban/peri-urban herds in Kampala and Gulu

Factor	*P-*value	Neonatal calf mortality rate (%) (Least Squares Mean ± S.E.M)
Education levels	0.069	
Primary level or No school		05.19 ± 0.01
Post primary education		10.63 ± 0.01
Record keeping	>0.15	
Yes		06.83 ± 0.01
No		08.99 ± 0.02
Herd size	0.0139	06.15 ± 0.02
≤3 dairy cows		09.67 ± 0.01
>3 dairy cows		
Record keeping * Production system interaction		
Records * Zero grazing	>0.15	08.35 ± 0.01
Records * Open grazing		05.31 ± 0.02
No records * Open grazing	>0.15	12.00 ± 0.04
No records * Zero grazing		05.97 ± 0.02
Type of farm * Dairy herd size interaction		
Commercial farming * ≤ 3 cows	0.0312	03.24 ± 0.03
Commercial farming * > 3 cows		11.16 ± 0.01
Subsistence or subsistence–commercial mix * ≤ 3 cows	>0.15	09.63 ± 0.03
Subsistence or subsistence–commercial mix * > 3 cows		08.18 ± 0.01

## Discussion

The primary objective of this study was to identify differences in socio-economic and animal husbandry factors between the capital Kampala and the northern municipality Gulu, and indeed the dairy farmers were more educated and had more experience in Kampala. As likely consequences, the milk production per cow and the calving rate were higher in Kampala than in Gulu. Also, in the overall analyses of data from the two locations, reproductive traits were associated with several socioeconomic, husbandry and production factors important to consider for interventions.

Among the socio-economic factors associated with dairy cow husbandry, it was found that Kampala farmers’ compliance with better keeping of farm records and infrastructural establishments to ease cow husbandry compared with the farmers in Gulu can be due to the two decade-long war and social unrest in the post-strife Gulu. A longer period of social stability may promote a market-led change in mindset for productive dairying [4, 6] and initiate specialized commercial production for many consumers [5].

The more zero grazing and smaller herds observed in Kampala compared with Gulu could probably be a result of a stronger human population pressure in the capital dictating conditions for UPU farming such as number of cows and access to grazing land [17, 18]. The dominance of dairy farming occupation in Kampala by males rather than females compared with Gulu was similarly observed in managerial decision-making study of households, in the UPU dairy farming system of Masaka, Uganda, [19]. The finding about fewer farmers belonging to institutionalized farmer-organizations in Kampala than Gulu, could be attributed to the fact that the Kampala farmers had a longer experience in dairy farming and felt no need for extension service, thus being more prone to an “do it your self-approach” for managing farming problems. Such attitude has been noted among pastoral communities in Kenya [20]. Related to this, it is somewhat surprising that the higher degree of belonging to institutionalized farmer-organizations in Gulu is not reflected in higher productivity and reproductive efficiency. Likely, the farming skills among Gulu farmers have not yet reached that of the Kampala farmers.

The more breeding of dairy cows in urban and peri-urban Kampala by artificial insemination than in Gulu could be attributed to higher farmer literacy, more commercial dairy farming engagement and better availability of artificial breeding services [21]. Dairy farming in Gulu being relatively new, still guided by rules set up the supporting farmer organizations whereas in Kampala it is not. Cases in Gulu must be reported to the extension workers provided by those organizations. This may explain why Kampala farmers tended to more frequently extract foetal membranes as early as 3 h after calving by farm personnel applying traditional pastoral practices.

Data from both locations was analyzed together in order to increase the size of the data set when trying to find the sources to variations in the reproductive traits. It was found that overall higher calving rates were associated with fewer cows per herd and higher milk production per cow. Similar effects were found in both locations as documented by the lack of interaction between those factors and location. This could be attributed to more care being given to individual cows in the small herds and that the high producing cows were provided better nutrition, housing, health service and management as shown in other studies [22]. A word of caution is though that the calving rate was higher in the farms that lacked proper records, which may indicate a wishful remembering among these farmers. Interestingly, the association between high milk production and high calving rate was more pronounced in the herds with shorter (two years or less) experience in farming, then among the more experienced farmers. To speculate this might be interpreted as one group of the new farmers are very committed and ambitious, resulting in high milk production and good calving rates. Further, the overall higher calving rates under open grazing system observed in this study could perhaps be attributable to better nutritional status, hygiene, welfare and heat expression as well as detection than in the zero grazing system.

The observation that abortion rates were overall higher among herds with late weaning (≥5 months) than in herds with early weaning (2–4 months), disagrees with the findings in a South African study [23] in smallholder herds where there was no association between abortion and time of calf weaning. Furthermore, abortion rates were lower in more extensive farms practicing open grazing, moreover with poor structural provisions for handling animals. This may seem contradictory, as open grazing animals are more likely to be exposed to cattle from other herds thereby increasing the risk for being infected by abortive pathogens. However, other causes of abortions like nutritional or toxic substances [24] may have influenced the findings in the current study.

The overall positive association between calf mortality and educational level among the cow owners in UPU livelihoods might be implicitly explained by shared multiple responsibilities between dairy farming and other occupations [17]. Subsequently, inadequate participation in calf care activity by multiple role-players has been suggested. The positive association between calf mortality and herd size may indicate a similar inability to take proper care of calves in these larger herds.

From a practical forward-looking perspective, the data may be used to better tailor the support of transition from self-subsistence dairy farming to commercial dairy farming. This could for instance be done by promoting farmer to farmer learning and empower farmers – via adequate veterinary services – to handle the critical aspects of successful dairy farming identified in this study.

## Conclusions

The study showed significant differences between Kampala and Gulu in reproductive performance and related husbandry factors for cows in the UPU dairy farming systems. We speculate in the socio-economic dissimilarity between these two Ugandan settings as explanation for the observed differences. In addition, in an overall analysis of the data for the reproductive performance traits calving and abortion rate and neonatal calf mortality we found associations with several husbandry and production traits. For some of these associations we suggest a causality, which in turn should be taken into account when providing advice to the UPU dairy farmers in the tropics.
